# Deep Learning-Based Early Warning Score for Predicting Clinical Deterioration in General Ward Cancer Patients

**DOI:** 10.3390/cancers15215145

**Published:** 2023-10-26

**Authors:** Ryoung-Eun Ko, Zero Kim, Bomi Jeon, Migyeong Ji, Chi Ryang Chung, Gee Young Suh, Myung Jin Chung, Baek Hwan Cho

**Affiliations:** 1 Department of Critical Care Medicine, Samsung Medical Center, School of Medicine, Sungkyunkwan University, 81 Irwon-ro, Gangnam-gu, Seoul 06351, Republic of Korea; ryoungeun.ko@samsung.com (R.-E.K.); icu.chung@samsung.com (C.R.C.); suhgy@skku.edu (G.Y.S.); 2 Medical AI Research Center, Samsung Medical Center, Seoul 06351, Republic of Korea; zero.kim@g.skku.edu (Z.K.); bomii.jeon@sbri.co.kr (B.J.); jimigyeong@gmail.com (M.J.); mj1.chung@samsung.com (M.J.C.); 3 Department of Data Convergence and Future Medicine, School of Medicine, Sungkyunkwan University, Seoul 06351, Republic of Korea; 4 Department of Medicine, Samsung Medical Center, Sungkyunkwan University, Seoul 06351, Republic of Korea; 5 Devision of Pulmonary and Critical Care Medicine, Department of Medicine, Samsung Medical Center, Sungkyunkwan University, Seoul 06351, Republic of Korea; 6 Department of Biomedical Informatics, School of Medicine, CHA University, Seongnam 13497, Republic of Korea; 7 Institute of Biomedical Informatics, School of Medicine, CHA University, Seongnam 13497, Republic of Korea

**Keywords:** artificial intelligence, clinical deterioration, early warning scores, deep learning, rapid response system

## Abstract

**Simple Summary:**

This study aimed to develop a new warning score for cancer patients who are at risk of getting worse in the hospital. Cancer patients can have serious problems due to their treatment or cancer itself. A quick response system is used to find and treat these patients. This study used a deep-learning method to create a warning score based on the changes in vital signs. The warning score, called Can-EWS, was better than the existing score, called MEWS, in predicting and preventing bad outcomes. Can-EWS also had fewer false alarms than MEWS. This study could help improve the safety and care of cancer patients in the hospital.

**Abstract:**

Background: Cancer patients who are admitted to hospitals are at high risk of short-term deterioration due to treatment-related or cancer-specific complications. A rapid response system (RRS) is initiated when patients who are deteriorating or at risk of deteriorating are identified. This study was conducted to develop a deep learning-based early warning score (EWS) for cancer patients (Can-EWS) using delta values in vital signs. Methods: A retrospective cohort study was conducted on all oncology patients who were admitted to the general ward between 2016 and 2020. The data were divided into a training set (January 2016–December 2019) and a held-out test set (January 2020–December 2020). The primary outcome was clinical deterioration, defined as the composite of in-hospital cardiac arrest (IHCA) and unexpected intensive care unit (ICU) transfer. Results: During the study period, 19,739 cancer patients were admitted to the general wards and eligible for this study. Clinical deterioration occurred in 894 cases. IHCA and unexpected ICU transfer prevalence was 1.77 per 1000 admissions and 43.45 per 1000 admissions, respectively. We developed two models: Can-EWS V1, which used input vectors of the original five input variables, and Can-EWS V2, which used input vectors of 10 variables (including an additional five delta variables). The cross-validation performance of the clinical deterioration for Can-EWS V2 (AUROC, 0.946; 95% confidence interval [CI], 0.943–0.948) was higher than that for MEWS of 5 (AUROC, 0.589; 95% CI, 0.587–0.560; *p* < 0.001) and Can-EWS V1 (AUROC, 0.927; 95% CI, 0.924–0.931). As a virtual prognostic study, additional validation was performed on held-out test data. The AUROC and 95% CI were 0.588 (95% CI, 0.588–0.589), 0.890 (95% CI, 0.888–0.891), and 0.898 (95% CI, 0.897–0.899), for MEWS of 5, Can-EWS V1, and the deployed model Can-EWS V2, respectively. Can-EWS V2 outperformed other approaches for specificities, positive predictive values, negative predictive values, and the number of false alarms per day at the same sensitivity level on the held-out test data. Conclusions: We have developed and validated a deep learning-based EWS for cancer patients using the original values and differences between consecutive measurements of basic vital signs. The Can-EWS has acceptable discriminatory power and sensitivity, with extremely decreased false alarms compared with MEWS.

## 1. Introduction

Recent advances in anticancer therapeutics and improved general care have led to increased survival in cancer patients [1]. However, cancer patients admitted to hospitals are at high risk of short-term deterioration due to treatment-related or cancer-specific complications [2]. Common complications that lead to clinical deterioration when transferred to the intensive care unit (ICU) and in-hospital death include community or nosocomial infections and organ-specific toxicities associated with the chemotherapy and sequelae of cancer, including bleeding, thrombosis, or tumor lysis syndrome [3,4,5,6]. Consequently, critically ill cancer patients account for up to 15% of all ICU patients [7,8,9].

Some hospitalized patients experience clinical deterioration associated with hospital mortality or unexpected ICU transfers, increasing morbidity and mortality [10,11]. These patients often show abnormal physiological signs before clinical deterioration [12]. Therefore, timely recognition of warning signs from deteriorating patients and appropriate treatment are critical for improving patient safety. Methods used to monitor patient health conditions, especially to detect emergencies or clinical changes early and help medical staff respond quickly, have been attempted in various ways, including NEWS (National Early Warning Score), NEWS2 (National Early Warning Score 2), and MC-EWS (Mayo Clinic Early Warning Score) [13]. These methods are primarily medical assessment tools focused on detecting changes in patient condition and providing early warning of emergencies in the medical field. These tools primarily assess a patient’s condition based on specific indicators, and RSS is a comprehensive system that integrates these tools to monitor the patient’s overall condition and quickly thrive in emergencies [14,15,16,17]. Rapid response systems (RRSs) have been widely used internationally, including specially trained staff members and systems, to respond to deteriorating hospitalized patients [18]. The association between early versus late ICU admission in cancer patients has been previously reported [19]. Delayed or unexpected admission to the ICU increases mortality in cancer patients [20,21].

RRSs are initiated when patients who are deteriorating or at risk of deteriorating are identified [22]. Vital sign-based early warning scores (EWSs) were developed and adopted for screening patients at high risk of clinical deterioration [10,11,18,23]. However, vital sign-based EWSs, already widely used in many hospitals for activating the RRS, were found to have poor discriminatory power for identifying cancer patients at risk of deterioration [24,25]. Additionally, some studies have expressed concerns about applying conventional scoring systems to cancer patients, as these track and trigger scores may not reflect the diverse patterns and rapid deterioration of these patients. Therefore, a disease-specific MEWS could potentially enhance its ability to detect these patients at risk.

Deep learning is the development of systems that can learn from and make predictions on data without the need to be explicitly programmed and is particularly useful in settings where signals and data are produced faster than the human brain can interpret [26]. Recently, several deep learning-based EWSs for predicting clinical deterioration in general ward patients have been developed and showed improved performance compared to conventional EWSs [27,28]. However, previous deep learning-based EWSs were created for general hospitalized patients and not verified in cancer patients. In addition, these deep learning-based EWSs used only original variables, which were also used for conventional EWSs.

In the present study, a deep learning-based EWS for cancer patients (Can-EWS) was developed using the original values, and the differences between consecutive measurements of variables and the performance were compared with modified EWS (MEWS) for predicting clinical deterioration in cancer patients.

## 2. Materials and Methods

### 2.1. Study Population

A retrospective cohort study was conducted on all oncology patients who were admitted to the general ward at Samsung Medical Center, a 1989-bed university-affiliated tertiary referral hospital in Seoul, South Korea, from January 2016 to December 2020. Patients 18 years of age or older and diagnosed with major oncologic malignancy were included in the study. Only the last admission was collected for patients with multiple hospital admissions during the study period. Patients who met the following criteria were excluded: (1) admission directly to the ICU at hospital admission, (2) admission for planned surgery, (3) no vital sign data recorded in the 24 h before in-hospital cardiac arrest (IHCA) or unexpected ICU transfer, or (4) only vital sign data <30 min recorded.

The institutional review board of the Samsung Medical Center approved this study and waived the requirement for informed consent due to the observational nature of the research. In addition, patient information was anonymized and de-identified before analysis.

### 2.2. Operation of the Medical Emergency Team

Details of the hospital RRS have been described in previous publications [29,30]. Because the hospital-wide medical emergency team (MET) at Samsung Medical Center was initiated at the beginning of March 2009, a set of MET activation criteria based on abnormal physiological variables is used by physicians and nurses, with a single criterion being sufficient to warrant calling the MET [30]. All physicians and nurses can directly contact the MET from wards using a dedicated phone number. In addition, an automated alert system was developed with the original MET activation process in August 2016. MEWS was automatically calculated in all ward patients when nurses entered the patient’s vital signs into the electronic medical chart, and MET was automatically activated. When activated, a MET is expected to arrive within 10 min and order diagnostic tests and treatments related to the patient’s condition. Following assessment and initial treatment, an individual plan is created for each patient, and a joint decision is made regarding whether to transfer the patient to the ICU.

### 2.3. Data Collection

Databases of vital signs were collected from consecutive hospitalized oncologic malignant patients. The data were extracted using our institution’s Clinical Data Warehouse (Darwin-C, Samsung Medical Center, Seoul, Republic of Korea), automatically retrieving data from electronic medical records. Five time-stamped basic vital signs, including systolic blood pressure (SBP), heart rate (HR), respiratory rate (RR), body temperature (BT), and level of consciousness, were collected during the hospitalization of the cancer patients. The exact time and location of event occurrences were also extracted from the electronic medical records. Levels of consciousness were collected using the AVPU scale (alert (A), response to voice (V), responses to pain (P), or unresponsive (U)) [31,32]. From the initially collected data, erroneous values considerably outside of the acceptable range of each vital sign (30–300 mm Hg of SBP, 40–120 mm Hg of DBP, 10–300 beats/min of HR, 3–60 breaths/min of RR, and 30–45 °C of BT) or non-numeric values were excluded and assumed to be missing values. Missing values were replaced with the most recent values measured before the time the missing value existed. The MEWS was calculated based on a previously published table [33]. International Classification of Diseases, Tenth Revision codes from prior admissions within 5 years were used to define major organ cancer.

The primary outcome was clinical deterioration, defined as the composite of IHCA and unexpected ICU transfer. IHCA was defined when cardiopulmonary resuscitation was performed during general ward admission based on the electronic medical record. Only the first event was used when cardiac arrest occurred several times during the hospital stay. Unexpected ICU transfer was an event in which patients were transferred to the medical ICU that did not originate from either the emergency department or the operating room [29,34].

### 2.4. Data Processing and Model Development

The data were divided into a training set (January 2016–December 2019) and a held-out test set (January 2020–December 2020). A stratified 10-fold cross-validation on the training set was used to determine the best hyper-parameters for the Can-EWS. Then, a deployed model was trained using the whole training set with the hyper-parameters and tested on the held-out test set. The five vital signs mentioned above were used (SBP, HR, RR, BT, and level of consciousness). Thus, input vectors were constructed of the five vital signs for every hourly time window. The objective of this study was to predict whether an input vector belonged to the event time window. The event time window was defined as the interval from 30 min to 24 h before clinical deterioration. Therefore, patients can have 24 vectors per day, and the prediction was made on every input vector. For boosting model performance, an additional five input variables were constructed using delta values (difference between consecutive measurements) for each vital sign. Thus, two models were developed: Can-EWS V1, which used input vectors of the original five input variables, and Can-EWS V2, which used input vectors of 10 variables (including an additional five delta variables). Both Can-EWS V1 and Can-EWS V2 were developed using a gated recurrent unit (GRU).

**Figure 1:**
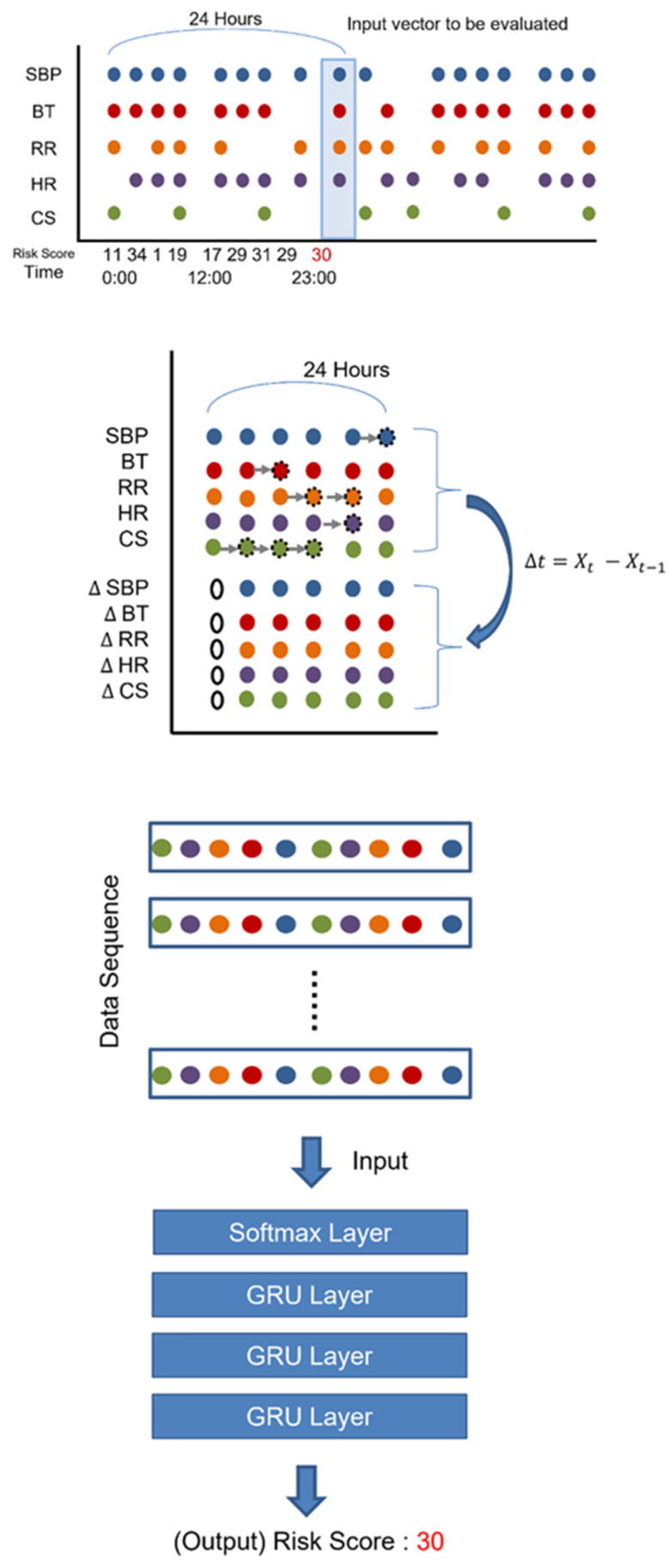
The process of Can-EWS. Can-EWS indicates the deep learning-based early warning system. SBP, systolic blood pressure; HR, heart rate; GRU, gated recurrent unit; RR, respiratory rate; BT, body temperature; CS, consciousness state; Δ, delta.

There are several effective models for dealing with time series data, including LSTM, GRU, and 1D CNN. The LSTM (Long Short-Term Memory) model is a type of recurrent neural network (RNN), which is good for learning time series patterns by managing long-term and short-term memory, and 1D CNN (Convolutional Neural Network with 1D Convolutions) can detect patterns in time series data as well. Among them, GRU has an effective and simpler structure (with fewer model parameters to learn) for managing long-term and short-term memory than LSTM, which makes it possible to learn time series patterns more efficiently with relatively less training data. After several trials to learn deep-learning models with LSTM and 1D CNN, we failed to train a converged model. Thus, we adopted GRU models in this study, consisting of three GRU layers with 24 neurons and a softmax layer at the output layer. We used the dropout rate of 0.3, the Adam optimizer with the default parameters, and a binary-cross entropy as a loss function (Figure 1).

Consequently, our automated early warning systems (Can-EWS V1 and V2) collect SBP, HR, RR, BT, and level of consciousness every hour from each patient. If there is any missing value for each variable, it is replaced with the most recently measured value. Also, additional delta variables are calculated if needed (for Can-EWS V2 only). Then, all the hourly measured variables are sequentially fed into the deep-learning models, which try to capture the time series pattern. Finally, based on the captured representation of the input time series data, our models calculate the early warning risk score as a probability between 0 and 100, as shown in Figure 1.

To calculate Can_EWS, we use the value that is closest to the calculation point among the values within 24 h. If there is no recorded value at that point, we use the most recent value instead. To calculate Can_EWS2, we added the delta value of the sorted values as a variable. Then, Can_EWS is calculated as a probability between 0 and 100 based on the captured representation of the input time series data.

### 2.5. Statistical Analysis

Data were presented as either median with interquartile range (IQR) or mean with standard deviation for continuous variables and number (%) for categorical variables. The discriminatory power of each score was assessed by calculating the area under the receiver operating characteristic (AUROC) curve. Prediction performance was also compared using sensitivity, specificity, positive predictive value (PPV), and negative predictive value (NPV). To calculate the AUROC, true-negative and false-positive rates were calculated in cancer patients who were not clinically deteriorating using each patient’s serial vital signs during their entire stay outside the ICU. For MEWS calculation, the highest scores for each patient during their hospitalization were used in clinically not deteriorated patients. The highest scores for each patient from 24 h to 30 min before IHCA or unexpected ICU transfer were used in clinically deteriorated patients. False-positive and true-negative rates were calculated using each patient’s serial vital signs from 24 h to 30 min before IHCA or unexpected ICU transfer in individuals who clinically deteriorated. Subsequently, the AUROC for primary and secondary outcomes was calculated based on the AUROC curve, and values for the estimated AUROC were compared using the McNeil test [35]. All analyses were performed using Python 3.6 TensorFlow and Keras.

## 3. Results

During the study period, 19,739 cancer patients were admitted to the general ward and had available medical records and documented vital signs. The mean age of the patients was 63.0 (±12.0) years, and the median length of hospital stay was 5.7 days. At admission, the initial mean SBP, HR, RR, and BT were 117.3 (±19.8) mmHg, 86.1 (±19.7) beats per min, 18.9 (±2.8) rates per min, and 36.6 °C (±0.5 °C), respectively. Among patients, 19,727 (99.8%) were alert at admission. Clinical deterioration occurred in 894 cases. IHCA and unexpected ICU transfer prevalence was 1.77 per 1000 admissions and 43.45 per 1000 admissions, respectively. The most common oncologic malignancy was lung cancer, followed by liver and biliary cancers.

### 3.1. Prediction Results Using 10-Fold Cross-Validation on the Training Set

**Figure 2:**
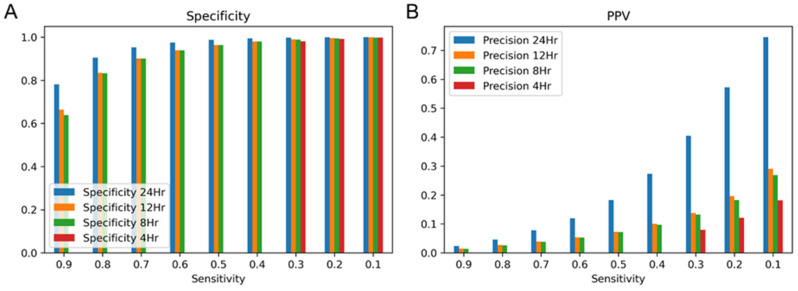
Specificity and PPV at fixed sensitivity levels along with different lookback period values for Can-EWS using 10-fold cross-validation. (**A**) Sensitivity and (**B**) PPV. PPV, positive predictive value.

The model’s performance was evaluated with various lookback periods from 4 to 24 h on the training data by 10-fold cross-validation. The lookback period can be considered as how much time the model needs to take a look at the vital sign sequences prior to now. Thus, the 4 h lookback period means the model takes the four latest input vectors to predict clinical deterioration. The model evaluation was made for four different lookback periods to calculate specificity and PPV at fixed sensitivity levels, as shown in Figure 2. When the lookback period was set to 24 h, the model showed the highest specificity and PPV at the highest sensitivity level. Therefore, the 24 h lookback period of sliding windows was utilized for further analysis and experiments.

**Figure 3:**
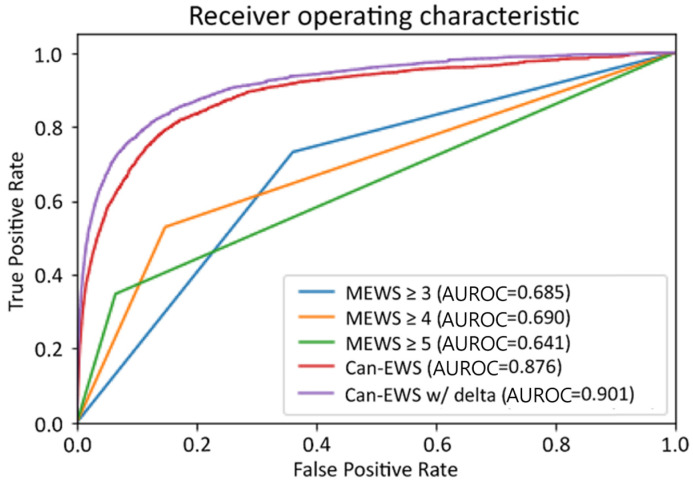
Receiver operating characteristics curve for predicting clinical deterioration. MEWS, modified early warning score; GRU, gated recurrent unit.

The AUROC of Can-EWS V1, Can-EWS V2, and MEWS with threshold scores 3, 4, and 5, which are widely accepted in clinical settings, were compared. As shown in Figure 3, the cross-validation performance of the clinical deterioration for Can-EWS V2 (AUROC. 0.946; 95% confidence interval [CI], 0.943–0.948) was higher than for MEWS of 3 (AUROC, 0.657; 95% CI, 0.656–0.658), 4 (AUROC, 0.645; 95% CI, 0.644–0.646), or 5 (AUROC, 0.589; 95% CI, 0.587–0.560; *p* < 0.001), and Can-EWS V1 (AUROC. 0.927; 95% CI, 0.924–0.931).

**Figure 4:**
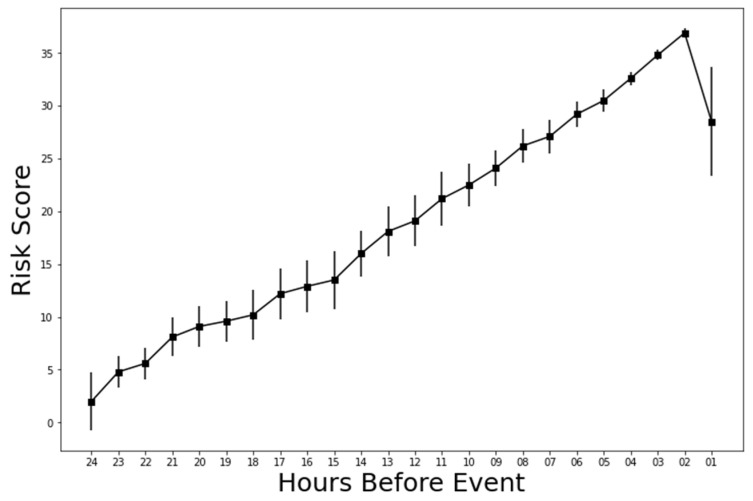
Average risk score for 24 h before clinical deterioration.

The risk score from the Can-EWS was also measured hourly. Because a softmax layer at the output of the Can-EWS was used, the probability of clinical deterioration could be estimated given the input vector sequence. Thus, the high-risk score (ranging from 0–100) means a high probability of clinical deterioration. The average of risk scores for 24 h before the actual clinical deterioration using the Can-EWS V2 and 24 h lookback is shown in Figure 4. Although the average risk score of all time for patients who did not undergo clinical deterioration remained at approximately 0.18 (not shown in Figure 4), the graph shows that as the patients get closer to their actual clinical deterioration, risk scores tend to get higher. The average risk score 24 h before substantial clinical damage was 3.11, which showed a significant difference from the average risk score of the cancer patient without clinical deterioration. Notably, after the highest average risk score (37.23) 2 h before deterioration, the risk score somewhat decreased 1 h before the actual clinical deterioration.

### 3.2. Additional Validation Using a Deployed Model on the Held-Out Test Data

For a virtual prognostic study, additional validation was performed on the held-out test data collected from 2019–2020 to evaluate further whether the trained model has generalization capability. The AUROC and 95% CI were 0.657 (95% CI, 0.656–0.657), 0.645 (95% CI, 0.644–0.645), 0.588 (95% CI, 0.588–0.589), 0.890 (95% CI, 0.888–0.891), and 0.898 (95% CI, 0.897–0.899), for MEWS of 3, 4, and 5, Can-EWS V1, and the deployed model Can-EWS V2, respectively. Table 1 and Table 2 show specificities, PPVs, NPVs, and the number of false alarms per day at the same sensitivity level for MEWS, Can-EWS V1, and Can-EWS V2. We summarized the sensitivity, PPV, NPV, and number of false alarms per day for Can-EWS V1 and Can-EWS V2, which can show the same sensitivity as MEWS 3, 4, and 5. The results showed that Can-EWS V2 outperformed other approaches for all measurements on the held-out test data.

## 4. Discussion

In the present study, Can-EWS, which uses the deep-learning algorithm with routinely collected vital signs, was shown to predict clinical deterioration in hospitalized cancer patients. Can-EWS V2, which used both original values and delta values (consecutive differences of original values), showed significant performance improvement compared with Can-EWS V1.

EWSs were developed based on the findings that abnormal changes in physiological parameters, such as vital signs and mental status, often precede overt clinical deterioration by several hours [36,37]. An alert is sent to the METs when the EWS exceeds a predetermined threshold. MEWS and its derivatives, such as the national EWS endorsed by the Royal College of Physicians for standard use across the United Kingdom, are already used by many hospitals as EWSs for RRSs [38,39]. MEWS includes SBP, HR, RR, BT, and level of consciousness, and values of 0–3 are given to each parameter based on the degree of abnormality [33]. Although clinical deterioration is characterized by physiological deterioration, there are limited data to support the suggestion that MEWS can predict outcomes among cancer patients [40]. In particular, Cooksly and colleagues reported that the MEWS has poor discriminatory values with AUROC 0.55 for identifying clinical deterioration in cancer patients [24].

Cancer patients are more severely ill than patients without cancer [41,42]. In addition, they often undergo short-term clinical deterioration associated with therapies for cancer and oncologic emergencies. In previous studies, an association was reported between early intervention and better outcomes despite the inherently severe condition of cancer patients [29,43]. Recently, major cancer centers have implemented RRSs along with advances in chemotherapy and trends of increasingly aggressive treatments [44]. Considering the unique characteristics of cancer patients, a different EWS might be needed to prevent clinical deterioration in these patients. Previously, several EWSs were developed for cancer patients. Lee JR and colleagues reported the MEWS plus SpO_2_/FiO_2_ score might be a helpful tool that can be used to identify clinical deterioration in hematological malignancies [45]. Young and colleagues showed the MEWS plus serum lactate was adequate for early identification of clinically deteriorating cancer patients [46]. In the present study, an EWS for cancer patients with variables in MEWS was developed using a deep learning-based method. Can-EWS V2 might be a novel EWS for cancer patients, and further external validation studies are needed.

Several deep learning-based EWSs have been reported and have shown acceptable performance [27,28,47]. These models adapted MEWS or National Early Warning Score variables and developed deep learning-based models. One strength of the present study was the use of both original and delta values. The additional delta information could be helpful for efficiently training the deep-learning models when the amount of training data is insufficient. Clinicians often include how the patient’s condition trends over time when assessing patients. Therefore, the delta value of the vital signs can potentially increase accuracy. The efficacy of the delta value was also reported in several previous studies. In a study from the USA, vital sign trends were evaluated, and delta PR and delta SBP were shown to predict the development of critical illness in general wards [48]. In a recent study from the UK, a new scoring system with vital signs, comorbidities, and frailty reportedly showed excellent performance. This model included dynamic variables and increased the model performance [49].

To use EWS in RRSs, detecting all clinically deteriorated patients is essential. However, having a minimal number of false alarms is also critical to reduce caregiver fatigue. Previous reports showed that alarm fatigue is an essential factor of the RRS operating barrier [50]. In this study, the number of false alarms per day with MEWS threshold 4 was 3.5 but was 0.8 for Can-EWS V1 and 0.5 for Can-EWS V2 at the same sensitivity as MEWS threshold 4. This result indicates the deep learning-based Can-EWS may improve the actual operation of RRSs.

In addition, the average risk score of clinical deterioration predicted by the Can-EWS V2 showed a gradual increase from 24 h before the actual event, whereas a flat low-risk score was shown for control cases. This pattern should be further investigated for use as another early warning signal. Notably, the risk score spontaneously decreased 1 h before clinical deterioration and requires further investigation through additional studies with more data. As shown in Table 1 and Table 2, the proper threshold for risk score is necessary for optimal outcomes. Therefore, using Can-EWS requires further prospective research to determine a suitable threshold.

The present study had several potential limitations that should be acknowledged. First, this study was retrospectively performed and validated. Prospective interventional studies are needed to verify the model’s performance and reconfirm its clinical usefulness. In addition, because Can-EWS was developed using patient data from a single institution, the generalizability of the findings requires further validation. However, a 10-fold held-out test was conducted for prospective study simulation. The training set and held-out test set represented the difference in data distribution (training set: 894 event cases, 18,875 control cases; held-out test set: 181 event cases, 5994 control cases), and this might result in a reduced PPV in the held-out test. Second, deep learning is considered a “black box” because it determines the relationship between the given data and a result and doesn’t create any explicit rule based on the captured knowledge. Therefore, additional efforts are needed to determine the reason for the alarm. Third, a maximum 24 h lookback period was used because a few patients were hospitalized for only 1 day, and whether testing with a longer lookback period improves predictive performance should be evaluated. Fourth, our dataset was highly imbalanced since positive cases for IHCA and unexpected ICU transfer were only 0.177% and 4.345% among the whole dataset. Therefore, in further studies, undersampling of the majority class or synthetic oversampling of the minority class with additional performance evaluation using PR-AUROC will be needed to tackle the class imbalance problem. Finally, we tried to compare the performances of our models with those developed from previous research [27] in Table 3, although the datasets are not the same between the two studies. It shows that the PPVs from our models are slightly higher than those of the previous study, whereas other measurements, such as sensitivity, specificity, and NPV, are almost the same. This implies that our model can potentially decrease the false positives and thus reduce unnecessary medical costs.

## 5. Conclusions

In the present study, we developed and validated Can-EWS, a deep-learning algorithm using routinely collected vital signs—including SBP, HR, RR, BT, and level of consciousness—that was shown to have acceptable performance for predicting clinical deterioration in hospitalized cancer patients and to outperform MEWS. Can-EWS V2, which used both original values and delta values, showed significant performance improvement compared with Can-EWS V1. Our data also suggest that Can-EWS produces appropriate alarms that lead to timely RRS intervention, highlighting its potential as an effective screening tool for detecting clinical deterioration in hospitalized cancer patients.

## Figures and Tables

**Table 1:** Prediction accuracy for clinical deterioration.

Score/Threshold	Sensitivity, %	Specificity, %	PPV, %	NPV, %	Number of False Alarms per Day
MEWS ≥ 3	73.1	63.8	1.3	99.7	8.7
Can-EWS V1 ≥ 0.011	73.1	94.2	6.8	99.8	0.74
Can-EWS V2 ≥ 0.013	73.1	97.0	12.1	99.8	0.4
MEWS ≥ 4	52.8	85.2	2.3	99.6	3.5
Can-EWS V1 ≥ 0.039	52.8	98.4	16.0	99.7	0.2
Can-EWS V2 ≥ 0.065	52.8	99.3	30.0	99.7	0.09
MEWS ≥ 5	34.7	93.5	3.5	99.5	1.3
Can-EWS V1 ≥ 0.130	34.7	99.6	33.2	99.6	0.05
Can-EWS V2 ≥ 0.221	34.7	99.8	55.0	99.6	0.2

MEWS, modified early warning score; PPV, positive predictive value; NPV, negative predictive value.

**Table 2:** Prediction accuracy for clinical deterioration using deploy model on the held-out test data.

Score/Threshold	Sensitivity, %	Specificity, %	PPV, %	NPV, %	Number of False Alarms per Day
MEWS ≥ 3	72.4	64.9	1.1	99.6	8.4
Can-EWS V1 ≥ 0.003	72.4	88.5	2.1	99.9	1.1
Can-EWS V2 ≥ 0.002	72.4	91.3	2.8	99.9	0.86
MEWS ≥ 4	53.2	85.8	2.0	99.7	3.5
Can-EWS V1 ≥ 0.013	53.2	96.3	4.8	99.8	0.36
Can-EWS V2 ≥ 0.006	53.2	97.0	5.7	99.8	0.3
MEWS ≥ 5	35.6	93.8	3.0	99.8	1.5
Can-EWS V1 ≥ 0.038	35.6	98.8	8.9	99.8	0.12
Can-EWS V2 ≥ 0.033	35.6	99.0	11.0	99.8	0.09

MEWS, modified early warning score; PPV, positive predictive value; NPV, negative predictive value.

**Table 3:** Comparison of DEWS and Can-EWS.

	Can-EWS V1	Can-EWS V2	DEWS *	MEWS *
Cohort	Single-center cancer patients	Single-center cancer patients	Two centers, all-cause admission	Two centers, all-cause admission
Number of patients	19,739	19,739	52,131	52,131
Years	2016–2020	2016–2020	2010–2017	2010–2017
Score	≥0.130	≥0.221	≥52.8	≥5
Sensitivity	34.7	34.7	37.3	37.3
Specificity	99.6	99.8	98.4	90.6
PPV	33.2	55.0	3.7	0.6
NPV	99.6	99.6	99.9	99.9

* The source of the data was a study by Kwon, J.M. et al. (2018) [27]. DEWS: deep learning-based early warning score; MEWS: modified early warning score; NPV: negative predictive value; PPV: positive predictive value.

## Data Availability

The data that support the findings of this study are available on request from the corresponding author. The data are not publicly available due to privacy or ethical restrictions.

## Abbreviations

AUROC	Area under the receiver operating characteristic
BT	Body temperature
Can-EWS	Deep learning-based EWS for cancer patients
EWS	Early warning score
GRU	Gated recurrent unit
HR	Heart rate
ICU	Intensive care unit
IHCA	In-hospital cardiac arrest
MET	Medical emergency team
MEWS	Modified early warning score
NPV	Negative predictive value
PPV	Positive predictive value
RR	Respiratory rate
RRS	Rapid response system
SBP	Systolic blood pressure

## References

[1] Allemani C., Matsuda T., Di Carlo V., Harewood R., Matz M., Niksic M., Bonaventure A., Valkov M., Johnson C.J., Esteve J. (2018). Global surveillance of trends in cancer survival 2000-14 (CONCORD-3): Analysis of individual records for 3,7513,025 patients diagnosed with one of 18 cancers from 322 population-based registries in 71 countries. Lancet.

[2] Yeo C.D., Kim J.W., Kim S.C., Kim Y.K., Kim K.H., Kim H.J., Lee S., Rhee C.K. (2012). Prognostic factors in critically ill patients with hematologic malignancies admitted to the intensive care unit. J. Crit. Care.

[3] Battaglia C.C., Hale K. (2019). Hospital-Acquired Infections in Critically Ill Patients with Cancer. J. Intensive Care Med..

[4] Kamboj M., Sepkowitz K.A. (2009). Nosocomial infections in patients with cancer. Lancet Oncol..

[5] Frere C., Font C., Esposito F., Crichi B., Girard P., Janus N. (2022). Incidence, risk factors, and management of bleeding in patients receiving anticoagulants for the treatment of cancer-associated thrombosis. Support Care Cancer.

[6] Rahmani B., Patel S., Seyam O., Gandhi J., Reid I., Smith N., Khan S.A. (2019). Current understanding of tumor lysis syndrome. Hematol. Oncol..

[7] Puxty K., McLoone P., Quasim T., Sloan B., Kinsella J., Morrison D.S. (2015). Risk of Critical Illness Among Patients with Solid Cancers: A Population-Based Observational Study. JAMA Oncol..

[8] Azoulay E., Moreau D., Alberti C., Leleu G., Adrie C., Barboteu M., Cottu P., Levy V., Le Gall J.R., Schlemmer B. (2000). Predictors of short-term mortality in critically ill patients with solid malignancies. Intensive Care Med..

[9] Taccone F.S., Artigas A.A., Sprung C.L., Moreno R., Sakr Y., Vincent J.L. (2009). Characteristics and outcomes of cancer patients in European ICUs. Crit. Care.

[10] Churpek M.M., Yuen T.C., Winslow C., Robicsek A.A., Meltzer D.O., Gibbons R.D., Edelson D.P. (2014). Multicenter development and validation of a risk stratification tool for ward patients. Am. J. Respir. Crit. Care Med..

[11] Escobar G.J., LaGuardia J.C., Turk B.J., Ragins A., Kipnis P., Draper D. (2012). Early detection of impending physiologic deterioration among patients who are not in intensive care: Development of predictive models using data from an automated electronic medical record. J. Hosp. Med..

[12] Buist M.D., Jarmolowski E., Burton P.R., Bernard S.A., Waxman B.P., Anderson J. (1999). Recognising clinical instability in hospital patients before cardiac arrest or unplanned admission to intensive care. A pilot study in a tertiary-care hospital. Med. J. Aust..

[13] Romero-Brufau S., Whitford D., Johnson M.G., Hickman J., Morlan B.W., Therneau T., Naessens J., Huddleston J.M. (2021). Using machine learning to improve the accuracy of patient deterioration predictions: Mayo Clinic Early Warning Score (MC-EWS). J. Am. Med. Inform. Assoc..

[14] Mohan S., Bhattacharya S., Kaluri R., Feng G., Tariq U. (2020). Multi-modal prediction of breast cancer using particle swarm optimization with non-dominating sorting. Int. J. Distrib. Sens. Netw..

[15] Zhang K., Zhang X., Ding W., Xuan N., Tian B., Huang T., Zhang Z., Cui W., Huang H., Zhang G. (2021). The prognostic accuracy of national early warning score 2 on predicting clinical deterioration for patients with COVID-19: A systematic review and meta-analysis. Front. Med..

[16] Adimoolam M., Govindharaju K., John A., Mohan S., Ahmadian A., Ciano T. (2021). A hybrid learning approach for the stage-wise classification and prediction of COVID-19 X-ray images. Expert Syst..

[17] Li D., Lyons P.G., Lu C., Kollef M. (2020). DeepAlerts: Deep learning based multi-horizon alerts for clinical deterioration on oncology hospital wards. Proc. AAAI Conf. Artif. Intell..

[18] Churpek M.M., Yuen T.C., Edelson D.P. (2013). Risk stratification of hospitalized patients on the wards. Chest.

[19] Azoulay E., Pène F., Darmon M., Lengliné E., Benoit D., Soares M., Vincent F., Bruneel F., Perez P., Lemiale V. (2015). Managing critically Ill hematology patients: Time to think differently. Blood Rev..

[20] Mokart D., Lambert J., Schnell D., Fouché L., Rabbat A., Kouatchet A., Lemiale V., Vincent F., Lengliné E., Bruneel F. (2013). Delayed intensive care unit admission is associated with increased mortality in patients with cancer with acute respiratory failure. Leuk. Lymphoma.

[21] Peigne V., Rusinová K., Karlin L., Darmon M., Fermand J.P., Schlemmer B., Azoulay E. (2009). Continued survival gains in recent years among critically ill myeloma patients. Intensive Care Med..

[22] Devita M.A., Bellomo R., Hillman K., Kellum J., Rotondi A., Teres D., Auerbach A., Chen W.J., Duncan K., Kenward G. (2006). Findings of the first consensus conference on medical emergency teams. Crit. Care Med..

[23] Umscheid C.A., Betesh J., VanZandbergen C., Hanish A., Tait G., Mikkelsen M.E., French B., Fuchs B.D. (2015). Development, implementation, and impact of an automated early warning and response system for sepsis. J. Hosp. Med..

[24] Cooksley T., Kitlowski E., Haji-Michael P. (2012). Effectiveness of Modified Early Warning Score in predicting outcomes in oncology patients. QJM Int. J. Med..

[25] Suhr K., Steen C., Albrecht-Thompson R., Williams J. (2020). NEWS Scoring System: Use in Hematologic Malignancies and Cellular Therapeutics Patient Populations. Clin. J. Oncol. Nurs..

[26] Shillan D., Sterne J.A.C., Champneys A., Gibbison B. (2019). Use of machine learning to analyse routinely collected intensive care unit data: A systematic review. Crit. Care.

[27] Kwon J.M., Lee Y., Lee Y., Lee S., Park J. (2018). An Algorithm Based on Deep Learning for Predicting In-Hospital Cardiac Arrest. J. Am. Heart Assoc..

[28] Shamout F.E., Zhu T., Sharma P., Watkinson P.J., Clifton D.A. (2020). Deep Interpretable Early Warning System for the Detection of Clinical Deterioration. IEEE J. Biomed. Health Inform..

[29] Song J.U., Suh G.Y., Park H.Y., Lim S.Y., Han S.G., Kang Y.R., Kwon O.J., Woo S., Jeon K. (2012). Early intervention on the outcomes in critically ill cancer patients admitted to intensive care units. Intensive Care Med..

[30] Na S.J., Ko R.E., Ko M.G., Koh A., Chung C.R., Suh G.Y., Jeon K. (2020). Risk Factors for Early Medical Emergency Team Reactivation in Hospitalized Patients. Crit. Care Med..

[31] American College of Surgeons, Committee on Trauma, Subcommittee on Advanced Trauma Life Support (1989). Advanced Trauma Life Support Course for Physicians.

[32] Kelly C.A., Upex A., Bateman D.N. (2004). Comparison of consciousness level assessment in the poisoned patient using the alert/verbal/painful/unresponsive scale and the Glasgow Coma Scale. Ann. Emerg. Med..

[33] Subbe C.P., Kruger M., Rutherford P., Gemmel L. (2001). Validation of a modified Early Warning Score in medical admissions. QJM Int. J. Med..

[34] Hillman K., Chen J., Cretikos M., Bellomo R., Brown D., Doig G., Finfer S., Flabouris A. (2005). Introduction of the medical emergency team (MET) system: A cluster-randomised controlled trial. Lancet.

[35] Hanley J.A., McNeil B.J. (1982). The meaning and use of the area under a receiver operating characteristic (ROC) curve. Radiology.

[36] Kause J., Smith G., Prytherch D., Parr M., Flabouris A., Hillman K. (2004). A comparison of antecedents to cardiac arrests, deaths and emergency intensive care admissions in Australia and New Zealand, and the United Kingdom--the ACADEMIA study. Resuscitation.

[37] Churpek M.M., Yuen T.C., Huber M.T., Park S.Y., Hall J.B., Edelson D.P. (2012). Predicting cardiac arrest on the wards: A nested case-control study. Chest.

[38] Smith G.B., Prytherch D.R., Meredith P., Schmidt P.E., Featherstone P.I. (2013). The ability of the National Early Warning Score (NEWS) to discriminate patients at risk of early cardiac arrest, unanticipated intensive care unit admission, and death. Resuscitation.

[39] Jones D.A., DeVita M.A., Bellomo R. (2011). Rapid-response teams. N. Engl. J. Med..

[40] Wise M.P., Barnes R.A., Baudouin S.V., Howell D., Lyttelton M., Marks D.I., Morris E.C., Parry-Jones N. (2015). Guidelines on the management and admission to intensive care of critically ill adult patients with haematological malignancy in the UK. Br. J. Haematol..

[41] Staudinger T., Stoiser B., Müllner M., Locker G.J., Laczika K., Knapp S., Burgmann H., Wilfing A., Kofler J., Thalhammer F. (2000). Outcome and prognostic factors in critically ill cancer patients admitted to the intensive care unit. Crit. Care Med..

[42] Austin C.A., Hanzaker C., Stafford R., Mayer C., Culp L., Lin F.C., Chang L. (2014). Utilization of rapid response resources and outcomes in a comprehensive cancer center. Crit. Care Med..

[43] Azoulay E., Soares M., Darmon M., Benoit D., Pastores S., Afessa B. (2011). Intensive care of the cancer patient: Recent achievements and remaining challenges. Ann. Intensive Care.

[44] Jones D., Warrillow S. (2014). Clinical deterioration in cancer patients--the role of the rapid response team. Crit. Care Med..

[45] Lee J.R., Jung Y.K., Kim H.J., Koh Y., Lim C.M., Hong S.B., Huh J.W. (2020). Derivation and validation of modified early warning score plus SpO2/FiO2 score for predicting acute deterioration of patients with hematological malignancies. Korean J. Intern. Med..

[46] Young R.S., Gobel B.H., Schumacher M., Lee J., Weaver C., Weitzman S. (2014). Use of the modified early warning score and serum lactate to prevent cardiopulmonary arrest in hematology-oncology patients: A quality improvement study. Am. J. Med. Qual..

[47] Kim J., Chae M., Chang H.J., Kim Y.A., Park E. (2019). Predicting Cardiac Arrest and Respiratory Failure Using Feasible Artificial Intelligence with Simple Trajectories of Patient Data. J. Clin. Med..

[48] Churpek M.M., Adhikari R., Edelson D.P. (2016). The value of vital sign trends for detecting clinical deterioration on the wards. Resuscitation.

[49] Pimentel M.A., Redfern O.C., Malycha J., Meredith P., Prytherch D., Briggs J., Young J.D., Clifton D.A., Tarassenko L., Watkinson P.J. (2021). Detecting Deteriorating Patients in Hospital: Development and Validation of a Novel Scoring System. Am. J. Respir. Crit. Care Med..

[50] Olsen S.L., Søreide E., Hillman K., Hansen B.S. (2019). Succeeding with rapid response systems—A never-ending process: A systematic review of how health-care professionals perceive facilitators and barriers within the limbs of the RRS. Resuscitation.

